# A Rare Case of Thoracoschisis

**DOI:** 10.21699/jns.v6i3.581

**Published:** 2017-08-10

**Authors:** Jamie Harris, Yanmin Zhang, Saurabh Patel, Benjamin Dille, Steven Garzon, Justin H. Lee, Bill Chiu

**Affiliations:** 1 Department of Surgery, Rush University Medical Center, 1750 W. Harrison St, Suite 785, Chicago, IL, 60612, USA; 2 Department of Pathology, University of Illinois at Chicago, 840 S. Wood Street, Suite 130, Chicago, IL 60612, USA; 3 Department of Neonatology, University of Illinois at Chicago, 840 S. Wood Street, Suite 1252, Chicago, IL, 60612, USA; 4 Department of Surgery, University of Illinois at Chicago, 840 S. Wood Street, Suite 416, Chicago, IL, 60612, USA

**Keywords:** Neonate, Exophytic liver, Thoracoschisis

## Abstract

A term male baby, after delivery, was found to have a 3-centimeter beefy-red mass protruding from the left chest wall, adjacent to the left nipple. Radiological imaging suggested it’s origin from the left lateral liver segment. A diagnostic laparoscopy confirmed the isolated connection to the liver, elevated left hemidiaphragm, and protrusion between the ribs. The mass was excised using electrocautery, and pathologic examination showed normal liver tissue.

## CASE REPORT

A male infant was delivered at 39 2/7 weeks gestation via spontaneous vaginal delivery and had APGAR scores of 9/9 with normal oxygen saturations on room air. Physical exam was notable for a left accessory nipple as well as a 3.3-centimeter exophytic mass at the midclavicular line on the left chest (Fig.1). This mass appeared nontender, irreducible, and beefy-red in color. No other abnormal physical exam findings were noted. Significant laboratory values included elevated total bilirubin of 7.1mg/dL at the time of birth and on repeat measurement decreased to 4.3mg/dL. Alpha fetoprotein (AFP) was 59,622 IU/mL, which was within the normal limits for the age. Chest radiograph demonstrated mild displacement of mediastinum to the right and the left hemidiaphram was elevated. An echocardiogram demonstrated normal cardiac structure. Abdominal ultrasound demonstrated normal hepatic blood flow without biliary dilation. An upper gastrointestinal swallow study delineated normal anatomy without any connection between this mass and the gastrointestinal tract lumen. Computerized tomography of the chest, abdomen and pelvis showed the mass likely originating from the liver with mediastinum shifted to the right, and a chest wall deformity at the site of the mass with normal number of ribs (Fig.1D,1E).

The mass progressively became enlarged, congested, and possibly ischemic over the course of three days. The base of the mass at the skin level was noted to be purple (Fig.1C). On day 4 of life, the patient was taken to the operating room. Initial laparoscopic inspection showed the mass arising from the left lateral lobe of the liver, extending to the left lateral chest wall and exiting between the ribs (Fig.2A,2B). Laparoscopy allowed us to clearly delineate the mass from the diaphragm before committing to laparotomy. The left hemidiaphragm was significantly elevated to the level of the mid-chest, but the rest of the abdominal content was otherwise normal. Traction placed on the exophytic portion of the mass confirmed the connection to the liver, and the chest wall opening had created a constrictive effect to demarcate the mass from the healthy liver. The mass was then separated from the liver using electrocautery (Fig.2C,2D). The fascia was closed where the mass had exited, and the skin opening was closed (Fig.2E).

**Figure:**
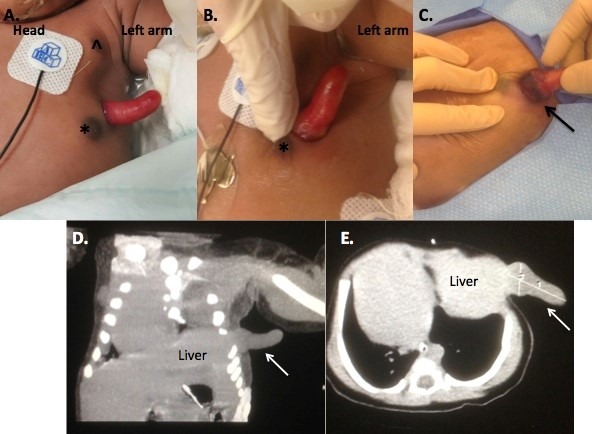
Figure 1: A. Appearance of the exophytic mass on initial examination. The mass was located on the left lateral chest between the normal (^) and accessory (*) left nipples. B. Upon close inspection, the mass was pink with a moist surface. C. Hyperemia and ischemia/necrosis at the base of the mass (black arrow) on day of life two. Coronal (D) and axial image (E) from computed tomography scan demonstrating extension of this mass from the liver (white arrow).

**Figure:**
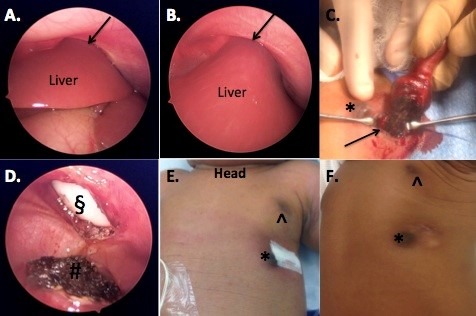
Figure 2: A and B. Laparoscopic images showing the left lateral segment of the liver in continuity with the external portion of the mass. The black arrow demonstrated the liver exiting through the chest wall. The left hemidiaphram was significantly elevated. C. Electrocautery was used to amputate the mass where it bordered the normal liver (black arrow). D. Post amputation of the exophytic mass demonstrated a circular chest wall defect (§), and hemostatic liver edge (#). E. Fascial closure and skin closure without further defect noted. Normal (^) and accessory left nipple (*) F. Three-month follow up demonstrating well-healed incision and mild chest wall deformity.

Postoperatively, chest radiograph demonstrated return of the mediastinum to midline and flattening of the left diaphragm to the expected anatomic location. Postoperative recovery was uneventful. Histologic examination of the specimen revealed normal liver tissue with ulceration and inflammation at the periphery. These findings were consistent with a thoracoschisis with normal liver herniating through the chest wall. At six-month follow up, the patient was clinically well and growing appropriately. The chest wall deformity had also improved. (Fig.2F).

## DISCUSSION

Thoracoschisis, which has been described as herniation of the abdominal contents through a chest wall defect, is an extremely rare congenital malformation. It was first described in 1977 in a patient that had both a diaphragmatic hernia and chest wall defect [1]. Prior reported literature described a female predominance of this condition [2,3]. Our case is the third occurrence described in a male. More frequently the defect is on the left side of the thorax. In all prior published cases, a diaphragmatic defect was identified [2,3], in contrast, our case was of an isolated thoracoschisis without diagrammatic defect.

The initial management of these patients began with assuring respiratory and hemodynamic stability. The patient may require supplemental oxygen after birth and feeds are held in case there was connection to the gastrointestinal tract. Our patient was clinically stable, which allowed us to get additional imaging. Subsequent workup such as x-ray, echocardiogram, ultrasound, computed tomography, and upper gastrointestinal series were aimed at distinguishing between the differential diagnoses. These studies concluded that this exophytic mass was likely originated from liver. Lesions such as hepatoblastoma, hamartoma, or hemangiomas were considered in differential diagnoses of nature of extruded mass, but ruled out on histopathology.

The mortality associated with the condition has been 50% in the literature, mostly due to the association with other malformations at birth. These included limb body wall complex and cardiac defects, neither of which were present in our case [4-7]. Our case demonstrates a less severe form of isolated thoracoschisis without diaphragmatic hernia. While the thoracoschisis did involve the chest wall in our patient, there was no evidence of hypoplasia or aplasia of any of the chest wall apart from that focal area. As respiratory complications can develop with chest wall deformities [8], long term follow up should be pursued.

## Footnotes

**Source of Support:**This work was supported by the National Institutes of Health grant R01NS094218 (to B.C.).

**Conflict of Interest:** None
